# Variable Frequency of Plastid RNA Editing among Ferns and Repeated Loss of Uridine-to-Cytidine Editing from Vascular Plants

**DOI:** 10.1371/journal.pone.0117075

**Published:** 2015-01-08

**Authors:** Wenhu Guo, Felix Grewe, Jeffrey P. Mower

**Affiliations:** 1 Center for Plant Science Innovation, University of Nebraska, Lincoln, Nebraska, United States of America; 2 School of Biological Sciences, University of Nebraska, Lincoln, Nebraska, United States of America; 3 ACGT, Inc., Wheeling, Illinois, United States of America; 4 Department of Agronomy and Horticulture, University of Nebraska, Lincoln, Nebraska, United States of America; NIH, UNITED STATES

## Abstract

The distinct distribution and abundance of C-to-U and U-to-C RNA editing among land plants suggest that these two processes originated and evolve independently, but the paucity of information from several key lineages limits our understanding of their evolution. To examine the evolutionary diversity of RNA editing among ferns, we sequenced the plastid transcriptomes from two early diverging species, *Ophioglossum californicum* and *Psilotum nudum*. Using a relaxed automated approach to minimize false negatives combined with manual inspection to eliminate false positives, we identified 297 C-to-U and three U-to-C edit sites in the *O. californicum* plastid transcriptome but only 27 C-to-U and no U-to-C edit sites in the *P. nudum* plastid transcriptome. A broader comparison of editing content with the leptosporangiate fern *Adiantum capillus-veneris* and the hornwort *Anthoceros formosae* uncovered large variance in the abundance of plastid editing, indicating that the frequency and type of RNA editing is highly labile in ferns. Edit sites that increase protein conservation among species are more abundant and more efficiently edited than silent and non-conservative sites, suggesting that selection maintains functionally important editing. The absence of U-to-C editing from *P. nudum* plastid transcripts and other vascular plants demonstrates that U-to-C editing loss is a recurrent phenomenon in vascular plant evolution.

## Introduction

In land plants (Embryophyta), plastid and mitochondrial transcripts undergo a type of post-transcriptional processing called RNA editing, which converts specific cytidines to uridines (C-to-U) or uridines to cytidines (U-to-C) through undefined mechanisms (reviewed in [1–3]). Surveys of organellar RNA editing have revealed extensive variability in the frequency and type of editing among and within the major land plant groups, which includes seed plants (Spermatophyta), ferns *sensu lato*(Monilophyta), lycophytes (Lycopodiophyta), hornworts (Anthocerotophyta), mosses (Bryophyta *sensu stricto*), and liverworts (Marchantiophyta). Numerous studies of RNA editing in seed plants (particularly angiosperms) have identified hundreds of C-to-U edit sites in the mitochondria and dozens of plastid C-to-U sites [4–8]. A few reports have suggested that U-to-C editing may also occur at a low frequency in some angiosperm mitochondria [9–12], although cloning or sequencing artifacts cannot be ruled out in these rare cases. Most moss and liverwort organelles have a low number of C-to-U sites and no U-to-C sites [13–15]. However, some early diverging lineages such as *Takakia* and *Haplomitrium* appear to have many C-to-U sites [16–18], while the complete absence of edited sites in marchantiid liverwort organellar transcripts indicates a secondary loss of editing in this group [16].

The pattern of RNA editing is more complex in hornworts, lycophytes, and ferns. Frequent C-to-U and U-to-C editing has been identified in plastid and mitochondrial transcripts from many hornworts [19–22]. Among lycophytes, both types of editing are apparent in *Isoetes* and *Huperzia* organelles, with pervasive editing in *Isoetes* mitochondria [23–26]. C-to-U editing is even more extensive in *Selaginella* organelles, whereas no U-to-C editing was found, indicating a secondary loss of U-to-C editing in this genus [27, 28]. The diversity of RNA editing is less clear in ferns. There is abundant C-to-U and U-to-C editing in the plastids of the leptosporangiate ferns *Adiantum capillis-veneris* and *Pteridium aquilinum* [29, 30]. In contrast, the absence of internal stop codons suggests a potential loss of plastid U-to-C editing in several early diverging ferns such as *Equisetum* and *Psilotum* [24, 31]. Analyses of individual mitochondrial genes indicate a similar pattern, in which U-to-C editing is clearly present in several ferns but absent (at least from the examined genes) in *Equisetum* and *Psilotum* [32–34].

The above results illustrate the differential distribution of C-to-U and U-to-C RNA editing among land plants, which implies independent origins and subsequent evolutionary trajectories of the two editing processes. C-to-U editing has been observed in the mitochondria and plastids of all major land plant groups but in none of the closely allied green algal lineages nor in marchantiid liverworts, indicating an origin in the common ancestor of land plants followed by a single loss of editing from marchantiid liverworts. In contrast, U-to-C editing appears to be confined to ferns, lycophytes, and hornworts. Most current hypotheses about land plant relationships indicate that hornworts are the sister group to vascular plants [35–37], suggesting that U-to-C editing originated in the common ancestor of vascular plants and hornworts, with independent losses from the lycophyte *Selaginella* and most (or all) seed plants. Several ferns may also lack U-to-C editing, but complete organellar transcriptomes are needed to substantiate this possibility. To begin to understand the evolutionary diversity of RNA editing among ferns, we sequenced the plastid transcriptomes of two early diverging species, the adder’s-tongue fern (*Ophioglossum californicum*) and the whisk fern (*Psilotum nudum*), using the Illumina platform.

## Materials and Methods

### RNA extraction and sequencing

*O. californicum* and *P. nudum* plants, which were previously sequenced to obtain the plastid genome sequences [38] (accession numbers KC117178 for *O. californicum* and KC117179 for *P. nudum*), were obtained from the living collection at the Beadle Center Greenhouse (University of Nebraska–Lincoln). For each species, an organelle-enriched RNA sample was prepared. Mature, above-ground tissue (50–100 g) was homogenized in a Waring blender, filtered through four layers of cheesecloth, and then filtered through one layer of Miracloth. The filtrate was centrifuged at 2500 × g in a Sorvall RC 6+ centrifuge, and then the supernatant was centrifuged at 12000 × g for 20 min to pellet organelles. RNA was isolated from the pellet using the RNeasy Plant RNA Kit (QIAGEN) and treated with DNase I (Fermentas) according to manufacturer’s instructions. Ribosomal RNA content was reduced from the enriched organellar RNA using the RiboMinus Plant Kit for RNA-Seq (Invitrogen) according to the supplied protocol.

The RiboMinus-treated organellar RNAs for both species were sent to the University of Nebraska Genomics Core Facility for 75 bp single-read sequencing on the Illumina GAII platform, generating 26.6 M raw reads for *O. californicum* and 23.6 M raw reads for *P. nudum*. Read quality was assessed using FastQC version 0.10.1 (http://www.bioinformatics.babraham.ac.uk/projects/fastqc/). Adapter and low-quality sequences were trimmed from the *O. californicum* and *P. nudum* raw reads using cutadapt version 1.4.1 (https://code.google.com/p/cutadapt/) with modified parameters (-a AGATCGGAAGAGC-q 20-m 35) before further analysis.

### An automated pipeline for edit site detection

All remaining data were mapped to the *O. californicum* and *P. nudum* plastid genomes using TopHat version 2.0.9 [39] with relaxed parameters (-N 4—read-gap-length 3—read-edit-dist 5 -I 5000—coverage-search). Transcription coverage maps were drawn from the TopHat mapping results in R v3.1.0 with both window-size and step-size of 10 bp, and the number of reads mapped per genomic region were calculated (S1 Table) using the BEDTools multicov command [40]. Mismatches were identified in the *O. californicum* and *P. nudum* plastid transcriptomes by comparing the mapped transcript reads to the reference genome sequences. To do so, a pileup mapping summary file was generated with the mpileup command in SAMtools version 0.1.19 [41]. All DNA:RNA mismatches were reported, including mismatches supporting putative C-to-U edit sites (seen as C:T mismatches for sense-strand genes and G:A mismatches for antisense-strand genes), mismatches supporting putative U-to-C edit sites (T:C and A:G), and all other types of mismatches attributable to some type of error rather than RNA editing (A:C, A:T, C:A, C:G, G:C, G:T, T:A, and T:G).

**Figure 1:**
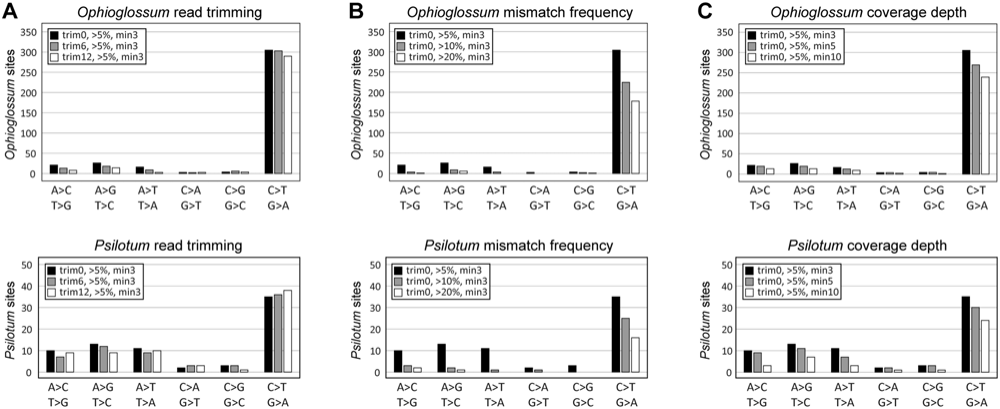
Results of automated detection of RNA edit sites with different cutoff values. All DNA:RNA mismatches have a minimum read depth of 3X and at least 5% of total reads and 3 individual reads support. A) Number of DNA:RNA mismatches recovered after no trimming (trim0), trimming all reads at both ends by 6 bp (trim6), or trimming all reads at both ends by 12 bp (trim12). B) Number of DNA:RNA mismatches supported by >5%, >10%, or >20% of total reads. C) Number of DNA:RNA mismatches supported with at least 3X, 5X or 10X read depth.

The read depth of the transcriptome mapping and the proportion of reads containing a mismatched nucleotide were calculated at each reference genome position by comparing the resulting pileup file with the reference plastid genome sequences. The pipeline required that all DNA:RNA mismatches have a minimum read depth of 3X and at least 5% of total reads or three individual reads supporting the mismatch, whichever is greater. Initial analysis on untrimmed data using these criteria (“trim0, >5%, min3” as black bars in Fig. 1) identified a large number of C:T and G:A mismatches, which is a clear signal of C-to-U editing in both species.

Additionally, the number of T:C and A:G mismatches was slightly higher than other mismatches, suggesting the possibility of U-to-C editing as well. However, there were also many identified mismatches that could not be caused by C-to-U or U-to-C editing, showing that many false positives were being identified by the automated approach.

Several modifications to the automated approach were evaluated in an attempt to reduce these false positive results (Fig. 1). To potentially eliminate false positives introduced by the higher rate of sequencing error and sequencing bias at the ends of reads (based on FastQC quality analysis), we compared the effect of trimming all reads by 6 bp or 12 bp at both ends (Fig. 1A). Although read trimming generally reduced the number of non-editing mismatches, it did not completely eliminate such sites. Furthermore, the different trimming treatments identified a slightly different set of C:T and G:A mismatches (due to slight differences in coverage at inefficiently edited sites that pushed the mismatch frequency just above or just below the 5% cutoff), raising the possibility that true edit sites could be missed from any single treatment, increasing false negatives. We also attempted to reduce the number of non-editing mismatches by increasing the minimum mismatch frequency threshold (Fig. 1B) or minimum depth-of-coverage threshold (Fig. 1C). While the number of non-editing mismatches dropped at higher thresholds, they were not eliminated completely. Furthermore, the increased stringency of these thresholds caused C:T and G:A mismatches (consistent to C-to-U editing) to drop much more dramatically, suggesting a substantial increase in false negatives at increased thresholds. These results demonstrated that there were no settings that could effectively reduce the number of false positives without also increasing the number of false negatives.

### Manual annotation of automated results to eliminate false positives

To minimize the number of false negatives with the automated approach, we retained all mismatches identified by all three read trimming treatments using the least stringent 5% mismatch frequency and 3x read depth thresholds. We then manually examined all mismatches by visually inspecting mapped reads using the SAMtools tview command. This inspection identified four factors that could provide alternative explanations for some DNA:RNA mismatches: 1) DNA heteroplasmy (i.e., sequence polymorphism among plastid genome copies), 2) errors in the transcriptome reads due to imperfect binding of random hexamers during cDNA preparation, as described previously [42, 43], 3) mapping artifacts at exon/intron junctions due to the mismapping of spliced transcript reads onto the unspliced reference genome sequence, and 4) errors in the reference genome sequence.

To eliminate false positives from the automated approach, we used a defined set of criteria to identify DNA:RNA mismatches arising by one of the alternative explanations defined above. Heteroplasmic regions in the genomes were detected by mapping Illumina DNA sequence data from the plastid genome sequencing projects [38] onto the plastid genome sequences using Bowtie version 2.2.2 [44] (S1 Fig). Mismatches introduced by imperfect primer binding were identified by searching for mismatches that occurred only within 6 bp of the end of all mapped transcript reads (S2 Fig). Mismatches near exon/intron junctions were inspected to determine if they resulted from the mismapping of spliced transcript reads onto the unspliced genome sequence (S3 Fig). Errors in the reference genomes were identified by mapping the Illumina DNA sequence data onto the plastid genome sequences using Bowtie (S4 Fig).

### Comparative analyses of RNA editing content, sequence effects, and editing efficiency

To compare editing content among species, genes from *O. californicum, P. nudum, Anthoceros formosae* (AB086179), and *Adiantum capillus-veneris* (AY178864) were aligned with clustalW [45] and manually adjusted when necessary. Homology of edit sites among species were determined based on the alignments. Using these alignments, the effect of RNA editing on the encoded amino acid were scored as conservative if editing improved amino acid identity with other species or non-conservative if editing did not improve or decreased amino acid identity with other species. Editing efficiency for each edit site was scored as the fraction of reads that contained the edited nucleotide sequence.

## Results

### An automated approach combined with manual inspection for edit site detection

To detect edit sites in the *O. californicum* and *P. nudum* plastid transcriptomes, we generated an automated bioinformatics pipeline that compared transcript reads to the plastid genome sequences that were previously sequenced from the same plants [38]. Although sequence mismatches consistent with RNA editing were most abundantly detected by this automated approach, it was clear that the pipeline also detected many false positive mismatches that could not be generated by RNA editing (Fig. 1), which implies that some of the editing-type mismatches may also be false positives. Modifications to the automated pipeline that altered the trimming of sequence reads (Fig. 1A), the minimal frequency threshold to detect mismatches (Fig. 1B), or the minimum depth of sequence coverage to examine (Fig. 1C) were insufficient to effectively eliminate all obvious false positives (i.e., the non-editing mismatches) without also introducing numerous unwanted false negatives (apparent by the even larger drop in the number of mismatches consistent with editing). Thus, none of the automated threshold settings could provide a satisfactory tradeoff between the number of false positives and false negatives. To address this issue, we set the automated thresholds to the lowest stringency to minimize the number of false negatives, and then we manually examined all mismatches to examine the potential sources of error and to eliminate false positives.

Manual inspection of results identified several sources of error that generated false positive signals. First, heteroplasmy (i.e., sequence polymorphism among plastid genome copies) occurring in transcribed segments of the genome produces polymorphism among transcripts, which generated a mismatch signal in our automated approach. To eliminate this issue, mismatches occurring at heteroplasmic sites (identified by mapping Illumina DNA reads onto the genome sequences) were removed from the results. Second, there was increased sequencing error at the ends of reads, mostly likely caused by imperfect binding of random hexamers during cDNA library preparation [42, 43]. To avoid this problem, we manually excluded all identified mismatches that were solely supported by reads where the mismatch occurred within 6 bp of the end. Third, several mismatches were caused by obvious mismapping of reads at exon/intron junctions and were also eliminated. Finally, there were a few sites where both DNA and RNA read mappings disagreed with the published genome sequence. Because the genomes and transcriptomes were sequenced from the same source plants, this discrepancy indicates an error in the genome sequence. These mismatches were removed from the results, and the genome sequence was corrected to the proper nucleotide. Altogether, our manual corrections eliminated all of the mismatches that could not be caused by C-to-U or U-to-C RNA editing, and also eliminated a small number of editing-type mismatches (S2 Table).

### Diverse types and frequency of plastid RNA editing among ferns

**Figure 2:**
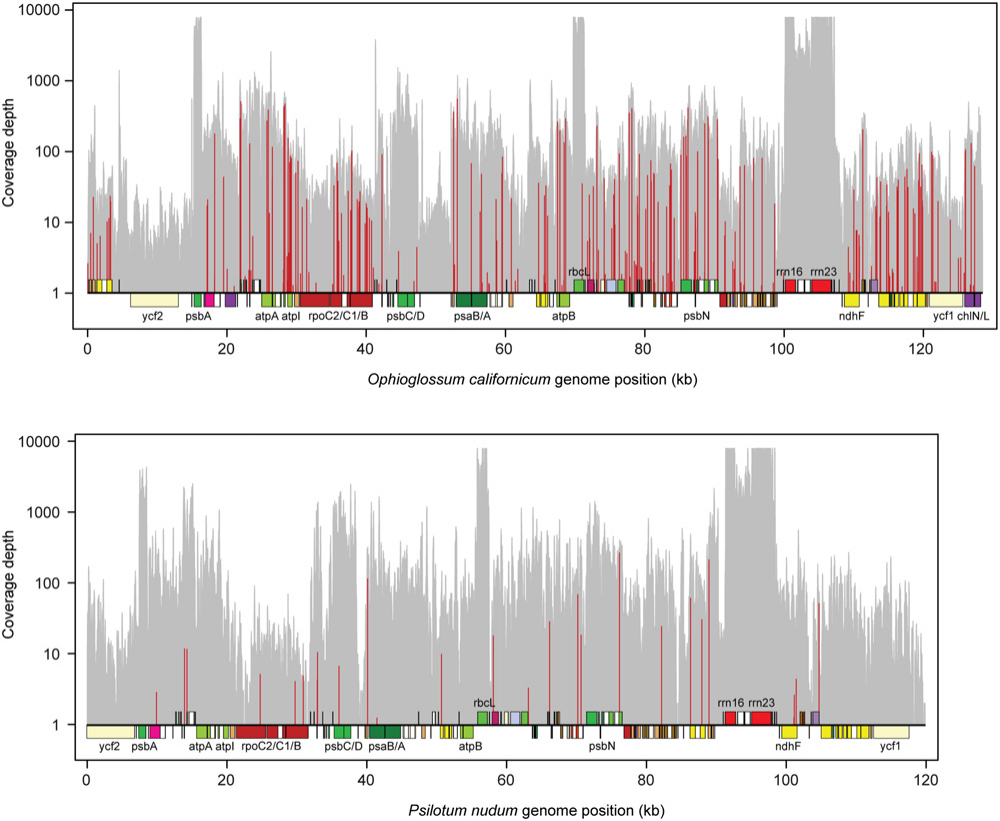
Transcriptome coverage depth and editing frequency. The *x*-axis shows the genome position and *y*-axis indicates read depth in logarithmic scale. For each genome, only one copy of the inverted repeat was shown. The histogram-like plot represents the transcriptome read depth. Red vertical lines show the genomic positions of edit sites, and the relative height of each red line compared with the corresponding read depth indicates editing efficiency.

**Figure 3:**
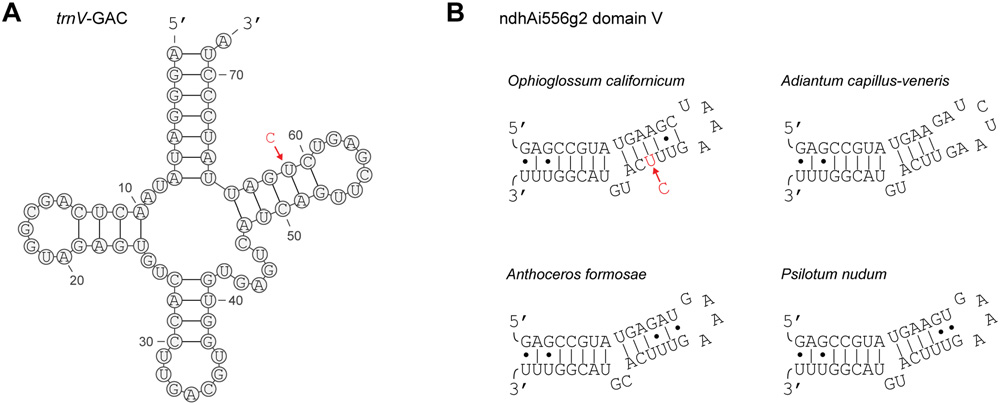
Structural RNA editing. RNA editing, shown in red, improves the stability of stem structures in A) the TψC arm of the tRNA *trnV*-GAC in *O. californicum* and B) domain V of the intron ndhAi556g2 in *O. californicum*, *A. capillus-veneris*, *A. formosae*, and *P. nudum*.

After manual inspection of mismatch results generated by the automated pipeline, a total of 297 C-to-U edit sites and three U-to-C edit sites were identified in the *O. californicum* plastid transcriptome (Fig. 2; S3 Table). The majority of sites (232/300) affect coding regions, and there is a strong preference for editing at second codon positions relative to first or third positions (Table 1). RNA editing alters the reading frame for eight genes, including the creation of five start codons (*accD*, *ndhA*, *ndhD*, *ndhF*, *psaB*) and two stop codons (*chIL* and *petD*) by C-to-U editing and the removal of an internal stop codon (*ycf1*) by U-to-C editing (Table 1). The *rps15* gene also appears to have a start codon created by editing, but the C-to-U change was supported by only a single transcript and was thus not included in our results. Outside of coding regions, a single edit site alters the *trnV*-GAC gene, which improves base pairing in the stem of the TψC arm (Fig. 3A). In addition, ten edit sites reside in six introns (clpPi71, clpPi363, ndhAi556, petBi6, rpoC1i432, and trnKUUUi37). At least one of these intronic edit sites, in domain V of intron ndhAi556, reconstructs an A:U base pairing that is conserved at the DNA level in related plants and appears essential for proper intron folding (Fig. 3B). The remaining 57 non-coding edit sites were found in intergenic regions, presumably affecting 5′ or 3′ UTRs. No edit sites were identified in any ribosomal RNAs.

**Table 1:** Number of edit sites in *O. californicum* and *P. nudum.*

	***O. californicum***		***P. nudum***	
Total	300	100.0%	27	100.0%
C-to-U	297	99.0%	27	100.0%
U-to-C	3	1.0%	0	0.0%
Coding	232	77.3%	24	88.9%
1st	26	8.7%	4	14.8%
2nd	163	54.3%	17	63.0%
3rd	43	14.3%	3	11.1%
start created	5	1.7%	0	0.0%
stop created	4	1.3%	0	0.0%
stop removed	1	0.3%	0	0.0%
Non-coding	68	22.7%	3	11.1%
intron	10	3.3%	1	3.7%
tRNA	1	0.3%	0	0.0%
UTR	57	19.0%	2	7.4%

In the *P. nudum* plastid transcriptome, a total of 27 C-to-U edit sites and no U-to-C sites were detected (Fig. 2; S3 Table), including the previously identified site in the *ndhB* gene [46]. Of these sites, 24 reside in coding regions and preferentially alter the second codon position, but no start or stop codons are created for any gene (Table 1). The three non-coding edit sites are located within intron clpPi71 and within the *psaM-ycf12* and *rpl21-rpl32* intergenic regions. No transfer RNAs or ribosomal RNAs were found to be edited.

**Figure 4:**
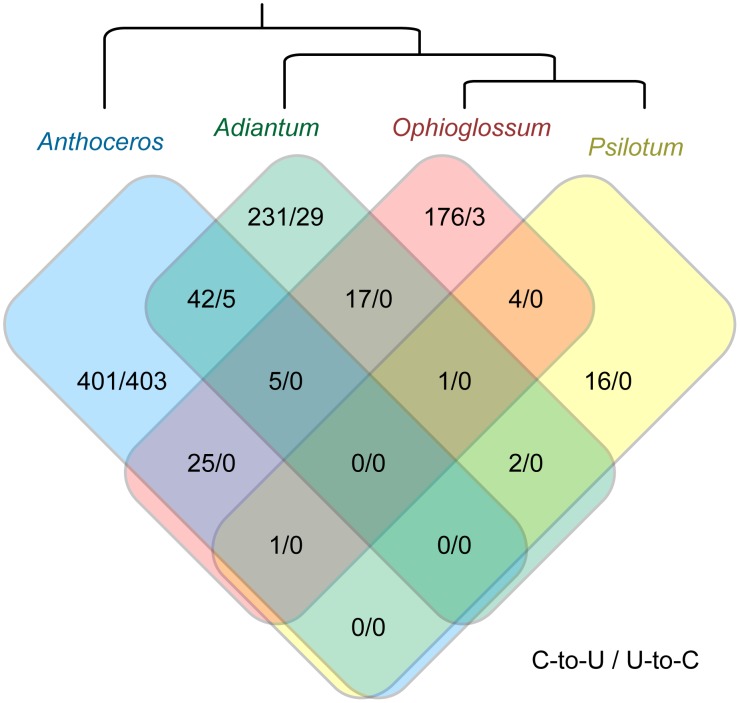
Comparison of plastid editing content among plants. The Venn diagram shows shared homologous C-to-U and U-to-C edit sites among coding regions of examined species. Non-coding edit sites were excluded from comparison.

A comparison of shared plastid RNA edit sites among the three ferns (*Adiantum capillus-veneris*, *O. californicum*, and *P. nudum*) and the hornwort outgroup (*Anthoceros formosae*) shows a large amount of variation in the frequency and type of RNA editing among species (Fig. 4). Of the >500 different edit sites identified in the coding regions of at least one fern, only 50 are shared among more than one fern or with the hornwort. That most of the edit sites are not shared among species indicates a highly lineage-specific pattern of frequent gain and loss during fern evolution. The presence of plastid U-to-C editing in two of the three examined ferns and the hornwort outgroup is most parsimoniously explained by the presence of this process in the common ancestor of all ferns followed by loss from the plastid of *P. nudum*.

### RNA editing efficiency correlates with functional effects and identifies potential mis-editing

**Figure 5:**
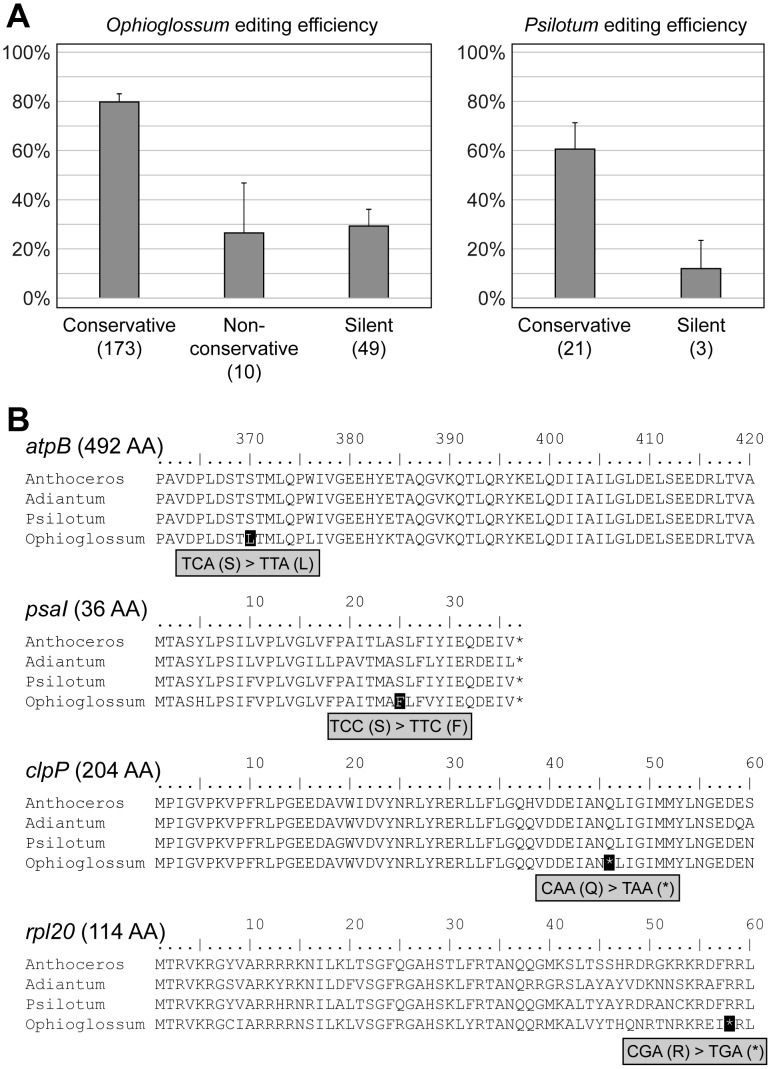
Relationship between functional effect and efficiency of editing. A) Column charts of overall editing efficiency for groups of RNA edit sites with different functional effects on the encoded amino acids. Error bars indicate two times the standard error. B) Multiple sequence alignments show the editing effect of several non-conservative RNA edit sites. The non-conservative edited position is shaded in black. The full length of each protein is listed in parentheses.

To examine the relationship between the functional effects of RNA editing on a protein sequence and the efficiency of editing in the plastid, we classified editing events in *O. californicum* and *P. nudum* protein-coding genes based on whether they improved sequence conservation to homologous proteins from other species (conservative sites), decreased conservation to homologous proteins (non-conservative sites), or did not alter the encoded amino acid (silent sites), and then we calculated editing efficiency as the proportion of reads that contained the edited nucleotide in the plastid transcriptome (Fig. 5A). Overall, edit sites that improve sequence conservation are substantially more abundant and more efficiently edited on average than non-conservative and silent editing events.

The rarity and generally low editing efficiency of the non-conservative sites in the *O. californicum* plastid transcriptome suggest that they result from mis-editing (Fig. 5B). For example, editing at position 1109 in the *atpB* gene changes a conserved serine to a leucine at only 5.1% efficiency (6/118 transcripts), while an edit site at position 74 in the *psaI* gene changes a conserved serine to a phenylalanine at 15% efficiency (41/267 transcripts). Both events substitute a small, hydrophilic amino acid with a large, hydrophobic amino acid, which may have negative effects on protein folding and function. In two more extreme cases, premature stop codons are introduced in the *clpP* gene at position 136 with 6.7% editing efficiency (5/75 transcripts) and in *rpl20* at position 172 with 6.0% editing efficiency (16/267 transcripts). These events, which truncate >75% of the CLPP protein and half of the RPL20 protein, would almost certainly abolish their functional activity.

## Discussion

**Figure 6:**
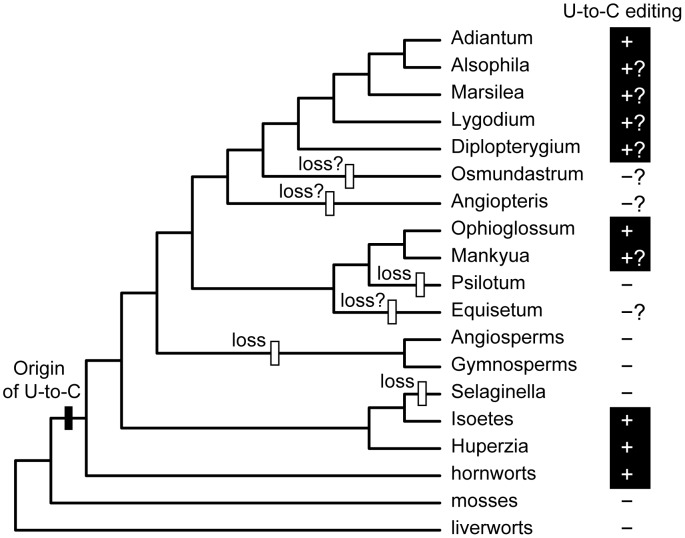
Distribution of U-to-C RNA editing among land plants. A plus sign indicates presence, a minus sign indicates absence. A question mark indicates that the presence or absence of U-to-C editing was inferred based on the presence or absence of internal stop codons in essential plastid genes. Inferred gain (black bar) and losses (white bars) of U-to-C editing were mapped using parsimony onto a phylogeny depicting the currently accepted relationships among land plants [35–37].

Our analyses have uncovered a large amount of variation in the frequency of C-to-U and U-to-C RNA editing among ferns. In addition, we demonstrated the complete loss of U-to-C editing from the plastid transcriptome of *P. nudum*. Together, these findings show that the frequency and type of RNA editing is highly labile in ferns. Importantly, the discovered loss of U-to-C editing from the *P. nudum* plastid transcriptome, along with the independent losses of U-to-C editing from seed plant and *Selaginella* plastids and potentially from additional fern lineages, reveals a recurrent pattern of loss of this process from vascular plants (Fig. 6). One caveat is that we cannot be absolutely certain that are no U-to-C edit sites in *P. nudum*. This same argument could also be made for the apparent absence of U-to-C editing from *Selaginella* and seed plant plastids. However, the overwhelming majority of the *P. nudum* plastid genome exhibits substantial transcription in our data set (Fig. 2), suggesting that few sites could have been missed due to low coverage. Furthermore, our analysis, which searched for very inefficiently edited sites (down to only 5% efficiency), is by far the most sensitive approach that has yet been performed for plastid RNA editing. Thus, while it is formally possible that a U-to-C edit site escaped detection because it is very inefficiently edited (<5% efficiency) or it is present in one of the very few lowly expressed (<3X depth of coverage) regions of the genome, we consider this to be unlikely.

The absence of any internal stop codons in many early diverging lineages suggests additional losses of U-to-C editing in ferns (Fig. 6). Because U-to-C editing often acts to eliminate internal stop codons in transcripts of essential genes, it is possible to predict the activity and relative abundance of U-to-C RNA editing in a species based on the presence and abundance of internal stop codons in otherwise intact and presumably functional genes, although it should be noted that the absence of internal stop codons does not guarantee that U-to-C editing is absent from the organelle. The available plastid genomes of most leptosporangiate ferns contain several internal stop codons in their genes [31], and the two sequenced plastid transcriptomes from *A. capillus-veneris* and *Pteridium aquilinum* confirm that U-to-C editing is abundant in these leptosporangiate ferns [29, 30]. In contrast, there are no internal stop codons in most early diverging fern lineages, including Psilotales, Equisetales, Marattiales, or in the earliest diverging lineage of leptosporangiate ferns, Osmundales [31], suggesting that U-to-C editing may be absent from these lineages. Here, we verified that the *P. nudum* plastid transcriptome lacks U-to-C editing, consistent with the absence of internal stop codons in its genome. More RNA editing analyses are needed, particularly from Equisetales, Marattiales, and Osmundales, to better understand the dynamic evolutionary history of C-to-U and U-to-C editing among ferns and to determine whether U-to-C editing was lost multiple times during fern evolution.

Our results also show a clear distinction in the frequency and efficiency of editing at sites that increase conservation among homologous proteins compared with sites that are silent or decrease sequence conservation, which is consistent with many previous observations in other vascular plant organelles (e.g., [4–8, 19, 25, 27–29, 47, 48–51]), indicating that this a general pattern of RNA editing across vascular plants. That conservative edit sites are more abundant and more efficiently edited strongly suggests that these sites are necessary for optimal protein function, and, furthermore, that selection is acting to maintain editing at these sites. Conversely, because silent editing events do not alter the protein sequence, there appears to be little to no selection at the protein level to retain these sites or to maintain the editing factors that control their editing efficiency. The extreme rarity of non-conservative editing suggests that selection is acting to eliminate many of these sites and reduce the editing efficiency of those that remain, thus mitigating their presumably negative consequences. The few non-conservative editing events that remain may occur at sites that are less functionally important in the protein, in which case the editing effects are selectively neutral and behave like silent editing events. Because we extracted RNA from all above-ground tissues, including stems and leaves in *O. californicum* and stems and enations in *P. nudum*, the low editing efficiency at some sites may reflect tissue-specific differences in editing efficiency, as shown in some plant organelles [12, 13, 52, 53]. Alternatively, it is possible that some of these silent and non-conservative events have a functional role, perhaps to regulate transcript stability, intron splicing efficiency, or the efficiency of editing at other sites in the transcript [47, 54, 55].

Edit sites in introns and intergenic regions are likewise rare and inefficiently edited on average, suggesting that most of these sites have no major function and are thus under little to no selective constraint to be maintained, similar to findings in other organellar transcriptomes [51, 56–58]. However, intronic editing in *O. californicum*(Fig. 3B) and other species [5, 19, 25, 59–62] appears in some cases to improve intron secondary structure, which is important to increase intron splicing efficiency. Intergenic edit sites in plant organellar transcripts may also have functional importance in some cases, such as regulating translational efficiency [7, 19, 63, 64].

Finally, our examination of false positives and false negatives revealed a wide variety of issues that can arise when using RNA-seq data to detect edit sites in a plant transcriptome. Overly stringent cutoff values introduced many false negatives by eliminating low-coverage and/or inefficiently edited sites from the automated results, while biological and technical issues such as heteroplasmy, imperfect binding of random hexamers during cDNA synthesis, mismapping at exon/intron junctions, and simple errors in the genome sequence generated a large number of false positive signals of editing that had to be eliminated. To minimize the number of false negatives, we used relaxed search parameters that included sites with low depth of coverage (down to only 3x read depth) and inefficient editing (down to only 5% efficiency), although we acknowledge that sites edited at less than 5% efficiency or covered by fewer than three transcript reads would have been missed by our approach. Because these relaxed parameter values increased the number of false positives, we manually evaluated all detected mismatches to eliminate signals due to biological and technical issues. In fact, our manual examination of results eliminated all of the mismatches that could not be caused by C-to-U or U-to-C RNA editing, verifying that our manual approach is able to efficiently detect and eliminate false positives. In summary, our work demonstrates that automated detection approaches alone are not sufficient for accurate editing detection. It is imperative to manually curate the automated results to ensure that false edit sites are not reported and true edit sites are not missed due to the issues described above. Additional suggestions, such as sequencing from organelle-enriched RNA, using DNase I to ensure no DNA contamination, priming with random hexamers rather than oligo-dT primers, subtracting rRNA from the RNA preparation, and employing sequence aligners (such as TopHat) that can handle mismatches and splicing junctions, were recently proposed to ensure optimal analysis of plant mitochondrial transcriptomes [65]. Many of these considerations are equally applicable to the sequencing of plant plastid transcriptomes and were implemented in our study.

## Supporting Information

S1 Fig False positive signals of RNA editing due to DNA heteroplasmy. The reference genome sequence is shown on the first line, the consensus mapping sequence is shown on the second line, and the remaining sequences are individually mapped reads. Numbers in the ruler indicate positions in the reference genome sequence.
(PDF)

S2 Fig False positive signals of RNA editing due to imperfect binding of random hexamers during cDNA preparation. (PDF)

S3 Fig False positive signals of RNA editing due to mapping artifacts at exon/intron junction sites. Shown at bottom is the unspliced reference genome sequence and the spliced RNA sequence.
(PDF)

S4 Fig False positive signals of RNA editing due to errors in the reference genome sequence. (PDF)

S1 Table Transcription read counts for different genomic locations in *O. californicum* and *P. nudum*. (XLS)

S2 Table Inferred false positive locations in (A) *O. californicum* and (B) *P. nudum*. (PDF)

S3 Table Edit site locations in (A) *O. californicum* and (B) *P. nudum*. (PDF)

## References

[1] Chateigner-Boutin AL, Small I (2011) Organellar RNA editing. Wiley Interdiscip Rev RNA 2: 493–506. 10.1002/wrna.72 21957039

[2] Finster S, Legen J, Qu Y, Schmitz-Linneweber C (2012) Land Plant RNA Editing or: Don’t Be Fooled by Plant Organellar DNA Sequences. In: Bock R Knoop V, editors. Genomics of Chloroplasts and Mitochondria: Springer Netherlands pp. 293–321.

[3] Takenaka M, Zehrmann A, Verbitskiy D, Hartel B, Brennicke A (2013) RNA editing in plants and its evolution. Annu Rev Genet 47: 335–352. 10.1146/annurev-genet-111212-133519 24274753

[4] Wakasugi T, Hirose T, Horihata M, Tsudzuki T, Kössel H, et al (1996) Creation of a novel protein-coding region at the RNA level in black pine chloroplasts: the pattern of RNA editing in the gymnosperm chloroplast is different from that in angiosperms. Proc Natl Acad Sci U S A 93: 8766–8770. 10.1073/pnas.93.16.8766 8710946 PMC38748

[5] Giegé P, Brennicke A (1999) RNA editing in *Arabidopsis* mitochondria effects 441 C to U changes in ORFs. Proc Natl Acad Sci U S A 96: 15324–15329. 10.1073/pnas.96.26.15324 10611383 PMC24818

[6] Grewe F, Edger PP, Keren I, Sultan L, Pires JC, et al (2014) Comparative analysis of 11 Brassicales mitochondrial genomes and the mitochondrial transcriptome of *Brassica oleracea* . Mitochondrion 19: 135–143. 10.1016/j.mito.2014.05.008 24907441

[7] Zeng WH, Liao SC, Chang CC (2007) Identification of RNA editing sites in chloroplast transcripts of *Phalaenopsis aphrodite* and comparative analysis with those of other seed plants. Plant Cell Physiol 48: 362–368. 10.1093/pcp/pcl058 17169923

[8] Chaw SM, Shih AC, Wang D, Wu YW, Liu SM, et al (2008) The mitochondrial genome of the gymnosperm *Cycas taitungensis* contains a novel family of short interspersed elements, Bpu sequences, and abundant RNA editing sites. Mol Biol Evol 25: 603–615. 10.1093/molbev/msn009 18192697

[9] Schuster W, Hiesel R, Wissinger B, Brennicke A (1990) RNA editing in the cytochrome *b* locus of the higher plant *Oenothera berteriana* includes a U-to-C transition. Mol Cell Biol 10: 2428–2431. 2325659 10.1128/mcb.10.5.2428-2431.1990 PMC360593

[10] Gualberto JM, Weil JH, Grienenberger JM (1990) Editing of the wheat *cox*III transcript: evidence for twelve C to U and one U to C conversions and for sequence similarities around editing sites. Nucleic Acids Res 18: 3771–3776. 10.1093/nar/18.13.3771 1695731 PMC331076

[11] Ong HC, Palmer JD (2006) Pervasive survival of expressed mitochondrial *rps14* pseudogenes in grasses and their relatives for 80 million years following three functional transfers to the nucleus. BMC Evol Biol 6: 55 10.1186/1471-2148-6-55 16842621 PMC1543663

[12] Picardi E, Horner DS, Chiara M, Schiavon R, Valle G, et al (2010) Large-scale detection and analysis of RNA editing in grape mtDNA by RNA deep-sequencing. Nucleic Acids Res 38: 4755–4767. 10.1093/nar/gkq202 20385587 PMC2919710

[13] Miyata Y, Sugita M (2004) Tissue- and stage-specific RNA editing of *rps14* transcripts in moss (*Physcomitrella patens*) chloroplasts. J Plant Physiol 161: 113–115. 10.1078/0176-1617-01220 15002671

[14] Rüdinger M, Funk HT, Rensing SA, Maier UG, Knoop V (2009) RNA editing: only eleven sites are present in the *Physcomitrella patens* mitochondrial transcriptome and a universal nomenclature proposal. Mol Genet Genomics 281: 473–481. 10.1007/s00438-009-0424-z 19169711

[15] Grosche C, Funk HT, Maier UG, Zauner S (2012) The chloroplast genome of *Pellia endiviifolia*: gene content, RNA-editing pattern, and the origin of chloroplast editing. Genome Biol Evol 4: 1349–1357. 10.1093/gbe/evs114 23221608 PMC3542565

[16] Groth-Malonek M, Wahrmund U, Polsakiewicz M, Knoop V (2007) Evolution of a pseudogene: exclusive survival of a functional mitochondrial *nad7* gene supports *Haplomitrium* as the earliest liverwort lineage and proposes a secondary loss of RNA editing in Marchantiidae. Mol Biol Evol 24: 1068–1074. 10.1093/molbev/msm026 17283365

[17] Yura K, Miyata Y, Arikawa T, Higuchi M, Sugita M (2008) Characteristics and prediction of RNA editing sites in transcripts of the Moss *Takakia lepidozioides* chloroplast. DNA Res 15: 309–321. 10.1093/dnares/dsn016 18650260 PMC2575889

[18] Rüdinger M, Volkmar U, Lenz H, Groth-Malonek M, Knoop V (2012) Nuclear DYW-type PPR gene families diversify with increasing RNA editing frequencies in liverwort and moss mitochondria. J Mol Evol 74: 37–51. 10.1007/s00239-012-9486-3 22302222

[19] Kugita M, Yamamoto Y, Fujikawa T, Matsumoto T, Yoshinaga K (2003) RNA editing in hornwort chloroplasts makes more than half the genes functional. Nucleic Acids Res 31: 2417–2423. 10.1093/nar/gkg327 12711687 PMC154213

[20] Duff RJ, Moore FB (2005) Pervasive RNA editing among hornwort *rbcL* transcripts except *Leiosporoceros* . J Mol Evol 61: 571–578. 10.1007/s00239-004-0146-0 16177870

[21] Li L, Wang B, Liu Y, Qiu YL (2009) The complete mitochondrial genome sequence of the hornwort *Megaceros aenigmaticus* shows a mixed mode of conservative yet dynamic evolution in early land plant mitochondrial genomes. J Mol Evol 68: 665–678. 10.1007/s00239-009-9240-7 19475442

[22] Xue JY, Liu Y, Li L, Wang B, Qiu YL (2010) The complete mitochondrial genome sequence of the hornwort *Phaeoceros laevis*: retention of many ancient pseudogenes and conservative evolution of mitochondrial genomes in hornworts. Curr Genet 56: 53–61. 10.1007/s00294-009-0279-1 19998039

[23] Wolf PG, Karol KG, Mandoli DF, Kuehl J, Arumuganathan K, et al (2005) The first complete chloroplast genome sequence of a lycophyte, *Huperzia lucidula* (Lycopodiaceae). Gene 350: 117–128. 10.1016/j.gene.2005.01.018 15788152

[24] Karol KG, Arumuganathan K, Boore JL, Duffy AM, Everett KD, et al (2010) Complete plastome sequences of *Equisetum arvense* and *Isoetes flaccida*: implications for phylogeny and plastid genome evolution of early land plant lineages. BMC Evol Biol 10: 321 10.1186/1471-2148-10-321 20969798 PMC3087542

[25] Grewe F, Herres S, Viehöver P, Polsakiewicz M, Weisshaar B, et al (2011) A unique transcriptome: 1782 positions of RNA editing alter 1406 codon identities in mitochondrial mRNAs of the lycophyte *Isoetes engelmannii* . Nucleic Acids Res 39: 2890–2902. 10.1093/nar/gkq1227 21138958 PMC3074146

[26] Liu Y, Wang B, Cui P, Li L, Xue JY, et al (2012) The mitochondrial genome of the lycophyte *Huperzia squarrosa*: the most archaic form in vascular plants. PLoS One 7: e35168 10.1371/journal.pone.0035168 22511984 PMC3325193

[27] Hecht J, Grewe F, Knoop V (2011) Extreme RNA editing in coding islands and abundant microsatellites in repeat sequences of *Selaginella moellendorffii* mitochondria: the root of frequent plant mtDNA recombination in early tracheophytes. Genome Biol Evol 3: 344–358. 10.1093/gbe/evr027 21436122 PMC5654404

[28] Oldenkott B, Yamaguchi K, Tsuji-Tsukinoki S, Knie N, Knoop V (2014) Chloroplast RNA editing going extreme: more than 3400 events of C-to-U editing in the chloroplast transcriptome of the lycophyte *Selaginella uncinata* . RNA 20: 1499–1506. 10.1261/rna.045575.114 25142065 PMC4174432

[29] Wolf PG, Rowe CA, Hasebe M (2004) High levels of RNA editing in a vascular plant chloroplast genome: analysis of transcripts from the fern *Adiantum capillus-veneris* . Gene 339: 89–97. 10.1016/j.gene.2004.06.018 15363849

[30] Wolf PG, Der JP, Duffy AM, Davidson JB, Grusz AL, et al (2011) The evolution of chloroplast genes and genomes in ferns. Plant Mol Biol 76: 251–261. 10.1007/s11103-010-9706-4 20976559

[31] Kim HT, Chung MG, Kim KJ (2014) Chloroplast genome evolution in early diverged leptosporangiate ferns. Mol Cells 37: 372–382. 10.14348/molcells.2014.2296 24823358 PMC4044308

[32] Hiesel R, Combettes B, Brennicke A (1994) Evidence for RNA editing in mitochondria of all major groups of land plants except the Bryophyta. Proc Natl Acad Sci U S A 91: 629–633. 10.1073/pnas.91.2.629 8290575 PMC43002

[33] Begu D, Araya A (2009) The horsetail *Equisetum arvense* mitochondria share two group I introns with the liverwort *Marchantia*, acquired a novel group II intron but lost intron-encoded ORFs. Curr Genet 55: 69–79. 10.1007/s00294-008-0225-7 19112563

[34] Sper-Whitis GL, Russell AL, Vaughn JC (1994) Mitochondrial RNA editing of cytochrome *c* oxidase subunit II (*coxII*) in the primitive vascular plant *Psilotum nudum* . Biochim Biophys Acta 1218: 218–220. 10.1016/0167-4781(94)90016-7 8018726

[35] Ruhfel BR, Gitzendanner MA, Soltis PS, Soltis DE, Burleigh JG (2014) From algae to angiosperms-inferring the phylogeny of green plants (Viridiplantae) from 360 plastid genomes. BMC Evol Biol 14: 23 10.1186/1471-2148-14-23 24533922 PMC3933183

[36] Qiu YL, Li L, Wang B, Chen Z, Knoop V, et al (2006) The deepest divergences in land plants inferred from phylogenomic evidence. Proc Natl Acad Sci U S A 103: 15511–15516. 10.1073/pnas.0603335103 17030812 PMC1622854

[37] Groth-Malonek M, Pruchner D, Grewe F, Knoop V (2005) Ancestors of *trans*-splicing mitochondrial introns support serial sister group relationships of hornworts and mosses with vascular plants. Mol Biol Evol 22: 117–125. 10.1093/molbev/msh259 15356283

[38] Grewe F, Guo W, Gubbels EA, Hansen AK, Mower JP (2013) Complete plastid genomes from *Ophioglossum californicum*, *Psilotum nudum*, and *Equisetum hyemale* reveal an ancestral land plant genome structure and resolve the position of Equisetales among monilophytes. BMC Evol Biol 13: 8 10.1186/1471-2148-13-8 23311954 PMC3553075

[39] Kim D, Pertea G, Trapnell C, Pimentel H, Kelley R, et al (2013) TopHat2: accurate alignment of transcriptomes in the presence of insertions, deletions and gene fusions. Genome Biol 14: R36 10.1186/gb-2013-14-4-r36 23618408 PMC4053844

[40] Quinlan AR, Hall IM (2010) BEDTools: a flexible suite of utilities for comparing genomic features. Bioinformatics 26: 841–842. 10.1093/bioinformatics/btq033 20110278 PMC2832824

[41] Li H, Handsaker B, Wysoker A, Fennell T, Ruan J, et al (2009) The Sequence Alignment/Map format and SAMtools. Bioinformatics 25: 2078–2079. 10.1093/bioinformatics/btp352 19505943 PMC2723002

[42] Hansen KD, Brenner SE, Dudoit S (2010) Biases in Illumina transcriptome sequencing caused by random hexamer priming. Nucleic Acids Res 38: e131 10.1093/nar/gkq224 20395217 PMC2896536

[43] van Gurp TP, McIntyre LM, Verhoeven KJ (2013) Consistent errors in first strand cDNA due to random hexamer mispriming. PLoS One 8: e85583 10.1371/journal.pone.0085583 24386481 PMC3875578

[44] Langmead B, Salzberg SL (2012) Fast gapped-read alignment with Bowtie 2. Nat Methods 9: 357–359. 10.1038/nmeth.1923 22388286 PMC3322381

[45] Larkin MA, Blackshields G, Brown NP, Chenna R, McGettigan PA, et al (2007) Clustal W and Clustal X version 2.0. Bioinformatics 23: 2947–2948. 10.1093/bioinformatics/btm404 17846036

[46] Freyer R, Kiefer-Meyer MC, Kössel H (1997) Occurrence of plastid RNA editing in all major lineages of land plants. Proc Natl Acad Sci U S A 94: 6285–6290. 10.1073/pnas.94.12.6285 9177209 PMC21041

[47] Mower JP, Palmer JD (2006) Patterns of partial RNA editing in mitochondrial genes of *Beta vulgaris* . Mol Genet Genomics 276: 285–293. 10.1007/s00438-006-0139-3 16862402

[48] Zehrmann A, van der Merwe JA, Verbitskiy D, Brennicke A, Takenaka M (2008) Seven large variations in the extent of RNA editing in plant mitochondria between three ecotypes of Arabidopsis thaliana. Mitochondrion 8: 319–327. 10.1016/j.mito.2008.07.003 18678284

[49] Sloan DB, MacQueen AH, Alverson AJ, Palmer JD, Taylor DR (2010) Extensive loss of RNA editing sites in rapidly evolving *Silene* mitochondrial genomes: selection vs. retroprocessing as the driving force. Genetics 185: 1369–1380. 20479143 10.1534/genetics.110.118000 PMC2927763

[50] Schuster W, Brennicke A (1991) RNA editing makes mistakes in plant mitochondria: editing loses sense in transcripts of a *rps19* pseudogene and in creating stop codons in *coxI* and *rps3* mRNAs of *Oenothera* . Nucleic Acids Res 19: 6923–6928. 10.1093/nar/19.24.6923 1762921 PMC329329

[51] Ruwe H, Castandet B, Schmitz-Linneweber C, Stern DB (2013) *Arabidopsis* chloroplast quantitative editotype. FEBS Lett 587: 1429–1433. 10.1016/j.febslet.2013.03.022 23523919

[52] Bentolila S, Elliott LE, Hanson MR (2008) Genetic architecture of mitochondrial editing in *Arabidopsis thaliana* . Genetics 178: 1693–1708. 10.1534/genetics.107.073585 17565941 PMC2278073

[53] Karcher D, Bock R (2002) The amino acid sequence of a plastid protein is developmentally regulated by RNA editing. J Biol Chem 277: 5570–5574. 10.1074/jbc.M107074200 11734554

[54] Li-Pook-Than J, Carrillo C, Niknejad N, Calixte S, Crosthwait J, et al (2007) Relationship between RNA splicing and exon editing near intron junctions in wheat mitochondria. Physiol Plant 129: 23–33. 10.1111/j.1399-3054.2006.00770.x

[55] Hepburn NJ, Schmidt DW, Mower JP (2012) Loss of two introns from the *Magnolia tripetala* mitochondrial *cox2* gene implicates horizontal gene transfer and gene conversion as a novel mechanism of intron loss. Mol Biol Evol 29: 3111–3120. 10.1093/molbev/mss130 22593225

[56] Carrillo C, Bonen L (1997) RNA editing status of *nad7* intron domains in wheat mitochondria. Nucleic Acids Res 25: 403–409. 10.1093/nar/25.2.403 9016571 PMC146442

[57] Carrillo C, Chapdelaine Y, Bonen L (2001) Variation in sequence and RNA editing within core domains of mitochondrial group II introns among plants. Mol Gen Genet 264: 595–603. 10.1007/s004380000345 11212914

[58] Richardson E, Dorrell RG, Howe CJ (2014) Genome-wide transcript profiling reveals the coevolution of plastid gene sequences and transcript processing pathways in the fucoxanthin dinoflagellate *Karlodinium veneficum* . Mol Biol Evol 31: 2376–2386. 10.1093/molbev/msu189 24925926 PMC4137713

[59] Börner GV, Mörl M, Wissinger B, Brennicke A, Schmelzer C (1995) RNA editing of a group II intron in *Oenothera* as a prerequisite for splicing. Mol Gen Genet 246: 739–744. 10.1007/BF00290721 7898443

[60] Castandet B, Choury D, Begu D, Jordana X, Araya A (2010) Intron RNA editing is essential for splicing in plant mitochondria. Nucleic Acids Res 38: 7112–7121. 10.1093/nar/gkq591 20615898 PMC2978366

[61] Farré JC, Aknin C, Araya A, Castandet B (2012) RNA editing in mitochondrial *trans*-introns is required for splicing. PLoS One 7: e52644 10.1371/journal.pone.0052644 23285127 PMC3527595

[62] Bégu D, Castandet B, Araya A (2011) RNA editing restores critical domains of a group I intron in fern mitochondria. Curr Genet 57: 317–325. 10.1007/s00294-011-0349-z 21701904

[63] Drescher A, Hupfer H, Nickel C, Albertazzi F, Hohmann U, et al (2002) C-to-U conversion in the intercistronic *ndhI*/*ndhG* RNA of plastids from monocot plants: conventional editing in an unconventional small reading frame? Mol Genet Genomics 267: 262–269. 10.1007/s00438-002-0662-9 11976970

[64] Grimes BT, Sisay AK, Carroll HD, Cahoon AB (2014) Deep sequencing of the tobacco mitochondrial transcriptome reveals expressed ORFs and numerous editing sites outside coding regions. BMC Genomics 15: 31 10.1186/1471-2164-15-31 24433288 PMC3898247

[65] Stone JD, Storchova H (2014) The application of RNA-seq to the comprehensive analysis of plant mitochondrial transcriptomes. Mol Genet Genomics: in press. 10.1007/s00438-014-0905-6 25182379

