# Supplementary material for: Variable Frequency of Plastid RNA Editing among Ferns and Repeated Loss of Uridine-to-Cytidine Editing from Vascular Plants
Source: PLoS One. 2015 Jan 8;10(1):e0117075. doi: 10.1371/journal.pone.0117075 (PMC4287625; doi:10.1371/journal.pone.0117075)

Psilotum RNA Read Mapping

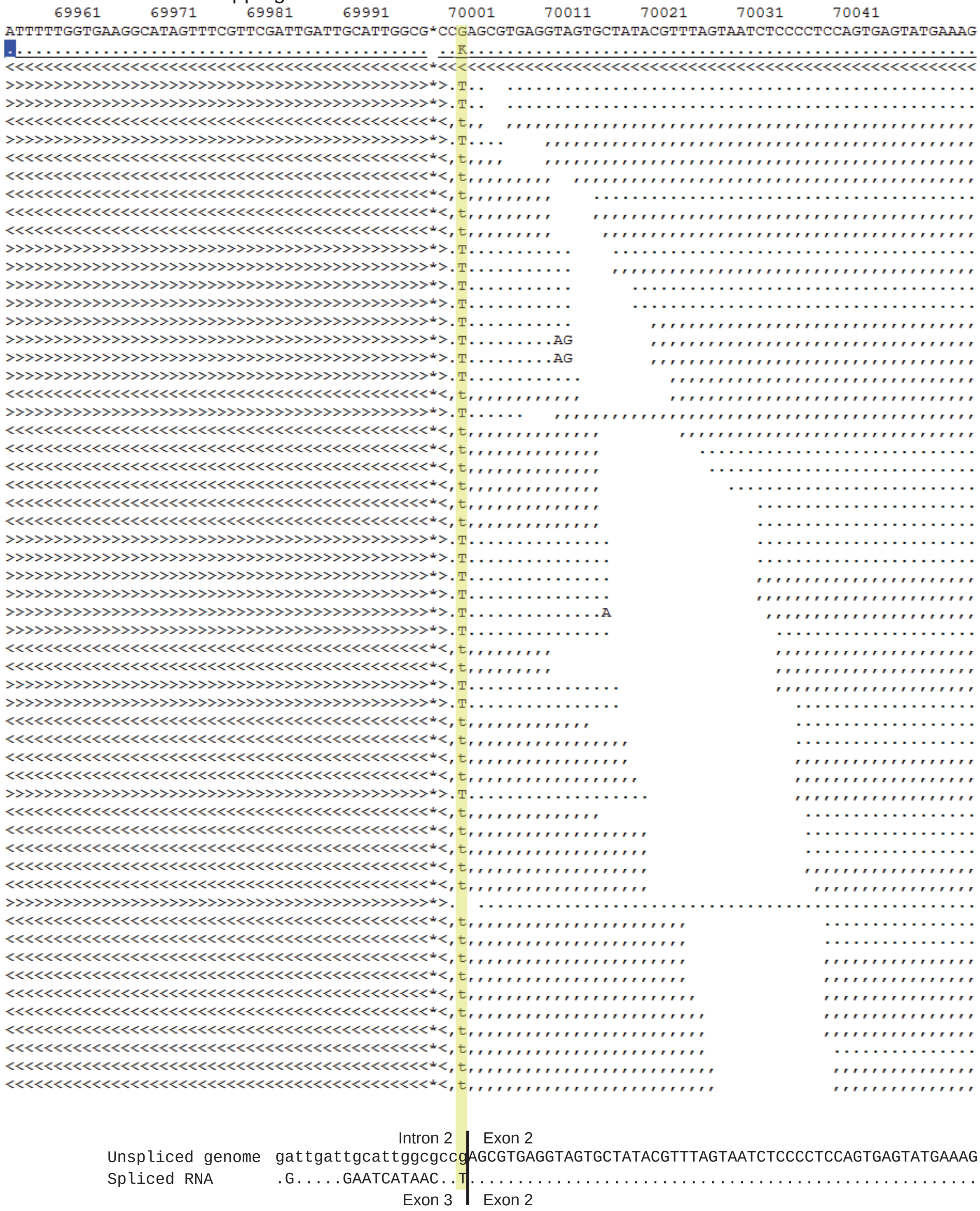

Supplement: S3 Fig — Shown at bottom is the unspliced reference genome sequence and the spliced RNA sequence. (PDF) [file pone.0117075.s003.pdf]
