# Supplementary material for: Variable Frequency of Plastid RNA Editing among Ferns and Repeated Loss of Uridine-to-Cytidine Editing from Vascular Plants
Source: PLoS One. 2015 Jan 8;10(1):e0117075. doi: 10.1371/journal.pone.0117075 (PMC4287625; doi:10.1371/journal.pone.0117075)
Supplement: S2 Table — (PDF) [file pone.0117075.s006.pdf]

Table S2A. Inferred false positive locations for *Ophioglossum californicum*

Genome Position	Ref Base	RNA Coverage	Mismatch Base	Mismatch Frequency	Inferred explanation for false positive
59	T	14	G	0.36	random hexamer induced sequencing error
60	T	15	A	0.33	random hexamer induced sequencing error
3502	A	47	G	0.13	random hexamer induced sequencing error
4494	T	99	G	0.07	random hexamer induced sequencing error
4495	T	105	C	0.06	random hexamer induced sequencing error
4504	A	480	G	0.10	random hexamer induced sequencing error
4958	C	24	T	0.62	DNA heteroplasmy
4961	A	24	C	0.62	DNA heteroplasmy
5512	T	31	C	0.13	DNA heteroplasmy
5517	A	52	C	0.40	DNA heteroplasmy
5521	A	57	G	0.42	DNA heteroplasmy
5586	T	44	C	0.64	DNA heteroplasmy
5590	T	32	C	0.56	DNA heteroplasmy
5595	T	26	C	0.46	DNA heteroplasmy
5604	T	28	C	0.36	DNA heteroplasmy
14616	T	20	A	0.15	random hexamer induced sequencing error
14618	T	20	A	0.15	random hexamer induced sequencing error
17738	T	35	G	0.14	random hexamer induced sequencing error
17741	T	32	C	0.16	random hexamer induced sequencing error
19482	T	53	A	0.17	random hexamer induced sequencing error
21518	C	85	A	0.06	random hexamer induced sequencing error
21780	G	97	C	0.05	random hexamer induced sequencing error
25173	T	287	G	0.06	random hexamer induced sequencing error
26347	A	1294	C	0.08	DNA heteroplasmy
28846	T	132	C	0.06	random hexamer induced sequencing error
28992	G	73	A	0.05	random hexamer induced sequencing error
30048	T	63	C	0.10	random hexamer induced sequencing error
30172	A	67	G	0.06	random hexamer induced sequencing error
32679	G	22	A	0.14	random hexamer induced sequencing error
38633	T	31	C	0.13	random hexamer induced sequencing error
41385	A	150	C	0.20	random hexamer induced sequencing error
44474	T	531	G	0.09	DNA heteroplasmy
45854	A	272	T	0.07	random hexamer induced sequencing error
45855	T	273	A	0.07	random hexamer induced sequencing error
46491	A	162	T	0.10	random hexamer induced sequencing error
46492	A	155	T	0.10	random hexamer induced sequencing error
46493	T	152	A	0.11	random hexamer induced sequencing error
47429	T	60	G	0.07	random hexamer induced sequencing error
50329	C	37	T	0.24	wrong strand
51121	G	17	C	1.00	reference genome error
53138	G	868	T	0.06	random hexamer induced sequencing error
54102	G	286	T	0.07	random hexamer induced sequencing error
55707	T	346	C	0.10	random hexamer induced sequencing error
58349	A	77	G	0.05	random hexamer induced sequencing error
59371	T	136	A	0.09	random hexamer induced sequencing error
59379	G	138	C	0.06	random hexamer induced sequencing error
62705	G	22	A	0.14	wrong strand
63082	G	6	A	0.67	wrong strand
66677	T	730	G	0.05	random hexamer induced sequencing error
67108	T	12	A	0.33	sequencing error
67194	G	30	A	0.10	random hexamer induced sequencing error
69143	A	112	G	0.06	random hexamer induced sequencing error
71513	G	44	A	0.07	wrong strand
72814	C	28	T	0.14	random hexamer induced sequencing error
77242	C	41	T	0.07	random hexamer induced sequencing error
82045	T	60	G	0.08	random hexamer induced sequencing error
87764	A	239	G	0.07	random hexamer induced sequencing error
89803	T	309	A	0.05	random hexamer induced sequencing error
93568	C	3	T	1.00	reference genome error
93570	T	4	C	0.75	reference genome error
96423	G	54	A	0.07	random hexamer induced sequencing error
97573	T	215	C	0.12	random hexamer induced sequencing error
99934	A	19	G	0.14	random hexamer induced sequencing error
100481	T	6414	A	0.05	rrn16, low mapping quality

100988	T	6842	G	0.10	rrn16, low mapping quality
100997	A	7081	C	0.14	rrn16, low mapping quality
101025	C	6734	T	0.19	rrn16, low mapping quality
101059	G	5747	A	0.19	rrn16, low mapping quality
101591	T	996	G	0.05	rrn16, low mapping quality
106398	G	612	A	0.19	rrn23, low mapping quality
106539	C	7610	T	0.10	rrn23, low mapping quality
106543	A	7461	C	0.08	rrn23, low mapping quality
107123	T	582	G	0.07	rrn5, low mapping quality
107888	A	83	G	0.81	random hexamer induced sequencing error
108039	G	21	C	0.24	random hexamer induced sequencing error
108040	T	20	A	0.25	random hexamer induced sequencing error
108041	A	20	C	0.25	random hexamer induced sequencing error
110105	C	76	T	0.05	random hexamer induced sequencing error
116518	T	85	C	0.13	wrong strand
117720	A	37	C	0.14	random hexamer induced sequencing error
119593	T	101	C	0.12	random hexamer induced sequencing error
122244	T	62	A	0.06	random hexamer induced sequencing error
127680	A	47	C	0.06	random hexamer induced sequencing error

Table S2B. Inferred false positive locations for *Psilotum nudum*

Genome Position	Ref Base	RNA Coverage	Mismatch Base	Mismatch Frequency	Interred explanation for false positive
2	C	5	T	1.00	mapping artifact, beginning of sequence
3	A	5	C	1.00	mapping artifact, beginning of sequence
4	A	5	C	1.00	mapping artifact, beginning of sequence
437	A	82	C	0.07	DNA heteroplasmy
6776	A	77	G	0.06	random hexamer induced sequencing error
8679	T	20	C	0.15	random hexamer induced sequencing error
9730	A	226	G	0.69	random hexamer induced sequencing error
10776	T	38	G	0.13	random hexamer induced sequencing error
13369	T	68	A	0.06	random hexamer induced sequencing error
17061	T	419	C	0.23	random hexamer induced sequencing error
17064	A	372	C	0.12	DNA heteroplasmy
21068	A	16	G	0.19	random hexamer induced sequencing error
35326	A	708	T	0.10	random hexamer induced sequencing error
35329	A	750	G	0.13	random hexamer induced sequencing error
35333	A	787	T	0.14	random hexamer induced sequencing error
40781	C	93	T	0.09	random hexamer induced sequencing error
40784	A	94	C	0.08	random hexamer induced sequencing error
44611	A	36	T	0.11	random hexamer induced sequencing error
51359	T	18	G	0.16	random hexamer induced sequencing error
59131	A	109	G	0.07	random hexamer induced sequencing error
59132	G	110	A	0.08	random hexamer induced sequencing error
59133	A	114	T	0.07	random hexamer induced sequencing error
60688	G	26	A	0.12	random hexamer induced sequencing error
62113	T	1142	A	0.15	random hexamer induced sequencing error
62117	G	1146	C	0.18	random hexamer induced sequencing error
62124	T	889	C	0.08	random hexamer induced sequencing error
63040	T	93	A	0.05	random hexamer induced sequencing error
65842	A	204	G	0.06	random hexamer induced sequencing error
66917	C	82	A	0.07	random hexamer induced sequencing error
68242	C	36	T	0.08	random hexamer induced sequencing error
70002	G	160	T	0.45	mapping artifact, exon/intron junction
73715	T	264	C	0.06	random hexamer induced sequencing error
80554	A	49	T	0.06	random hexamer induced sequencing error
86160	T	93	G	0.05	random hexamer induced sequencing error
86161	T	91	C	0.05	random hexamer induced sequencing error
86162	G	92	C	0.05	random hexamer induced sequencing error
86164	A	98	T	0.05	random hexamer induced sequencing error
87043	G	119	C	0.07	mapping artifact, exon/intron junction
91640	A	6202	G	0.08	rrn16, low mapping quality
92190	C	4124	T	0.15	rrn16, low mapping quality
92219	C	2567	T	0.08	rrn16, low mapping quality
92224	G	1250	A	0.16	rrn16, low mapping quality
97669	C	5377	T	0.12	rrn23, low mapping quality
97673	A	5932	C	0.09	rrn23, low mapping quality
98656	A	42	G	0.17	random hexamer induced sequencing error
100346	T	52	G	0.21	random hexamer induced sequencing error
104333	T	6	A	0.50	random hexamer induced sequencing error
116276	T	49	A	0.14	random hexamer induced sequencing error
