# Supplementary material for: Variable Frequency of Plastid RNA Editing among Ferns and Repeated Loss of Uridine-to-Cytidine Editing from Vascular Plants
Source: PLoS One. 2015 Jan 8;10(1):e0117075. doi: 10.1371/journal.pone.0117075 (PMC4287625; doi:10.1371/journal.pone.0117075)
Supplement: S3 Table — (PDF) [file pone.0117075.s007.pdf]

Table S3A. Plastid editing locations for *Ophioglossum californicum*

Genome Position	Ref Base	RNA Coverage	Editing Efficiency	Location	Codon Position	Codon Change	AA Change	Edit Site Class
28	C	7	0.714	intergenic: 5' of rps12i2	–	–	–	–
36	C	4	0.750	intergenic: 5' of rps12i2	–	–	–	–
441	C	43	0.512	rps12	2	TCA->TTA	S->L	Conservative
834	C	71	0.732	rps7	2	TCA->TTA	S->L	Conservative
1372	C	20	0.550	ndhB	2	TCA->TTA	S->L	Conservative
1807	C	10	0.700	ndhB	2	ACG->ATG	T->M	Conservative
1816	C	10	0.800	ndhB	2	TCA->TTA	S->L	Conservative
2792	C	15	0.800	ndhB	2	TCC->TTC	S->F	Conservative
2795	C	15	0.667	ndhB	2	TCA->TTA	S->L	Conservative
3064	C	27	0.741	ndhB	1	CAC->TAC	H->Y	Conservative
3251	C	40	0.850	ndhB	2	CCC->CTC	P->L	Conservative
3323	C	36	0.861	ndhB	2	CCT->CTT	P->L	Conservative
17150	G	113	0.593	matK	2	ACT->ATT	T->I	Conservative
17240	G	307	0.521	matK	2	TCG->TTG	S->L	Conservative
18234	G	291	0.086	intron: trnKUUUi37	–	–	–	–
18236	G	291	0.911	intron: trnKUUUi37	–	–	–	–
19529	G	82	0.866	intergenic: 5' of trnK-UUU, 3' of chlB	–	–	–	–
19532	G	85	0.718	intergenic: 5' of trnK-UUU, 3' of chlB	–	–	–	–
20038	G	38	0.211	chlB	3	CCC->CCT	P->P	Silent
21046	G	57	0.053	chlB	3	ATC->ATT	I->I	Silent
21889	C	933	0.827	psbK	2	TCG->TTG	S->L	Conservative
22003	C	484	0.994	psbK	2	CCA->CTA	P->L	Conservative
22081	C	118	0.059	intergenic: 5' of psbI, 3' of psbK	–	–	–	–
22611	C	215	0.098	intergenic: 5' of psbI, 3' of trnS-CGA	–	–	–	–
23151	C	197	0.142	intergenic: 5' of ycf12, 3' of trnS-GCU	–	–	–	–
23278	G	826	0.712	psaM	2	TCT->TTT	S->F	Conservative
23461	C	119	0.151	intergenic: 5' of ycf12	–	–	–	–
23723	C	354	0.311	ycf12	2	TCA->TTA	S->L	Conservative
25751	G	281	1.000	atpA	2	TCT->TTT	S->F	Conservative
25946	G	372	0.995	atpA	2	TCG->TTG	S->L	Conservative
26113	G	577	0.412	atpA	3	CTC->CTT	L->L	Silent
26517	G	160	0.731	intergenic: 5' of atpA, 3' of atpF	–	–	–	–
26530	G	132	0.955	intergenic: 5' of atpA, 3' of atpF	–	–	–	–
28103	G	229	0.061	intergenic: 3' of atpH	–	–	–	–
28135	G	483	0.124	intergenic: 3' of atpH	–	–	–	–
28168	G	487	0.975	atpH	2	CCA->CTA	P->L	Conservative
28327	G	421	0.998	atpH	2	CCT->CTT	P->L	Conservative
28634	G	28	0.357	intergenic: 5' of atpH, 3' of atpI	–	–	–	–
28652	G	32	0.125	intergenic: 5' of atpH, 3' of atpI	–	–	–	–
28783	G	235	0.932	atpI	2	CCA->CTA	P->L	Conservative
28869	G	106	0.280	atpI	3	CTC->CTT	L->L	Silent
29014	G	63	1.000	atpI	2	TCA->TTA	S->L	Conservative
29020	G	65	0.985	atpI	2	TCA->TTA	S->L	Conservative
29131	G	110	0.945	atpI	2	TCA->TTA	S->L	Conservative
29253	G	88	0.068	atpI	3	ACC->ACT	T->T	Silent
29260	G	75	0.987	atpI	2	TCA->TTA	S->L	Conservative
29502	G	135	0.052	intergenic: 5' of atpI, 3' of rps2	–	–	–	–
29890	G	83	0.880	rps2	2	GCT->GTT	A->V	Conservative
29912	A	67	0.060	rps2	1	TTA->CTA	L->L	Silent
30214	G	142	0.859	rps2	2	TCA->TTA	S->L	Conservative
30556	G	26	0.192	rpoC2	3	CAC->CAT	H->H	Silent
30819	G	18	0.944	rpoC2	1	CCA->TCA	P->S	Conservative
30823	G	20	0.500	rpoC2	3	TTC->TTT	F->F	Silent
31459	G	24	0.417	rpoC2	3	TTC->TTT	F->F	Silent
31460	G	24	0.958	rpoC2	2	TCC->TTC	S->F	Conservative
31769	G	15	0.267	rpoC2	2	TCG->TTG	S->L	Nonconservative

32803	G	28	0.107	rpoC2	3	TTC->TTT	F->F	Silent
35191	G	57	0.070	rpoC1	3	TTC->TTT	F->F	Silent
35372	G	51	0.882	rpoC1	2	CCC->CTC	P->L	Conservative
35807	G	101	0.911	rpoC1	2	TCT->TTT	S->F	Conservative
35940	G	64	0.862	rpoC1	1	CGT->TGT	R->C	Conservative
35962	G	41	0.667	rpoC1	3	TTC->TTT	F->F	Silent
36080	G	39	0.475	rpoC1	2	TCA->TTA	S->L	Conservative
36437	G	18	0.889	rpoC1	2	TCA->TTA	S->L	Conservative
36758	G	92	0.130	intron: rpoC1i432	–	–	–	–
37410	G	59	0.814	rpoC1	2	TCT->TTT	S->F	Conservative
37778	G	13	1.000	rpoB	2	CCG->CTG	P->L	Conservative
37907	G	152	0.901	rpoB	2	CCA->CTA	P->L	Conservative
37908	G	154	0.617	rpoB	1	CTA->TTA	L->L	Silent
37943	G	221	0.864	rpoB	2	TCT->TTT	S->F	Conservative
38717	G	61	0.738	rpoB	2	CCA->CTA	P->L	Conservative
38930	G	25	0.920	rpoB	2	TCT->TTT	S->F	Conservative
38978	G	43	0.791	rpoB	2	TCG->TTG	S->L	Conservative
38984	G	40	0.800	rpoB	2	TCA->TTA	S->L	Conservative
39107	G	39	0.897	rpoB	2	TCA->TTA	S->L	Conservative
39839	G	12	0.917	rpoB	2	TCT->TTT	S->F	Conservative
40016	G	29	0.862	rpoB	2	ACA->ATA	T->I	Conservative
40046	G	15	0.733	rpoB	2	ACA->ATA	T->I	Conservative
40055	G	12	0.917	rpoB	2	TCA->TTA	S->L	Conservative
40118	G	28	0.536	rpoB	2	CCT->CTT	P->L	Conservative
40136	G	25	0.840	rpoB	2	CCG->CTG	P->L	Conservative
40160	G	26	0.731	rpoB	2	TCG->TTG	S->L	Conservative
40451	G	29	0.759	rpoB	2	CCG->CTG	P->L	Conservative
40781	G	20	0.800	rpoB	2	TCT->TTT	S->F	Conservative
41016	G	23	0.174	intergenic: 5' of rpoB	–	–	–	–
41023	G	23	0.261	intergenic: 5' of rpoB	–	–	–	–
41834	C	306	0.052	intergenic: 3' of petN	–	–	–	–
42206	G	142	0.056	intergenic: 3' of psbM	–	–	–	–
42211	G	142	0.056	intergenic: 3' of psbM	–	–	–	–
42245	G	80	0.337	psbM	2	CCC->CTC	P->L	Conservative
42254	G	97	0.526	psbM	2	TCA->TTA	S->L	Conservative
42257	G	103	0.631	psbM	2	TCT->TTT	S->F	Conservative
42272	G	274	0.854	psbM	2	CCA->CTA	P->L	Conservative
42278	G	316	0.769	psbM	2	TCC->TTC	S->F	Conservative
44654	G	449	0.223	psbC	3	GCC->GCT	A->A	Silent
46860	G	176	0.074	psbD	3	TTC->TTT	F->F	Silent
47283	G	277	0.264	intergenic: 5' of psbD	–	–	–	–
52535	G	379	0.987	rps14	2	TCG->TTG	S->L	Conservative
52691	G	193	0.175	intergenic: 5' of rps14	–	–	–	–
53077	G	607	0.979	psaB	2	CCG->CTG	P->L	Conservative
55096	G	109	0.891	psaB	2	ACG->ATG	T->M	Conservative
56362	G	64	0.641	psaA	3	CTC->CTT	L->L	Silent
56561	G	53	0.962	psaA	2	TCT->TTT	S->F	Conservative
56652	G	46	0.087	psaA	1	CTG->TTG	L->L	Silent
57379	G	171	0.129	intergenic: 5' of psaA	–	–	–	–
57590	G	267	0.056	intergenic: 5' of psaA, 3' of ycf3	–	–	–	–
57666	G	258	0.050	intergenic: 5' of psaA, 3' of ycf3	–	–	–	–
58642	G	179	0.587	ycf3	2	TCG->TTG	S->L	Conservative
58753	G	62	0.403	ycf3	2	TCA->TTA	S->L	Conservative
59473	G	116	0.819	ycf3	2	TCT->TTT	S->F	Conservative
59551	G	86	0.953	ycf3	2	TCT->TTT	S->F	Conservative
60842	G	37	0.811	rps4	2	TCA->TTA	S->L	Conservative
61413	G	19	0.158	intergenic: 5' of rps4	–	–	–	–
61755	G	38	0.211	intergenic: 5' of rps4	–	–	–	–
64753	G	69	0.275	ndhJ	3	TTC->TTT	F->F	Silent
64754	G	68	0.853	ndhJ	2	TCC->TTC	S->F	Conservative

65527	G	38	0.158	ndhK	3	TTC->TTT	F->F	Silent
65597	G	26	0.962	ndhK	2	CCT->CTT	P->L	Conservative
65678	G	67	0.075	ndhK	2	CCT->CTT	P->L	Conservative
65788	G	51	0.882	ndhC	2	CCA->CTA	P->L	Conservative
66050	G	19	0.842	ndhC	1	CGG->TGG	R->W	Conservative
67474	G	507	0.892	atpE	2	ACT->ATT	T->I	Conservative
67800	G	217	0.991	atpB	2	TCA->TTA	S->L	Conservative
68112	G	118	0.051	atpB	2	TCA->TTA	S->L	Nonconservative
68393	G	152	0.086	atpB	3	TTC->TTT	F->F	Silent
68415	G	148	0.980	atpB	2	TCT->TTT	S->F	Conservative
68419	G	146	0.575	atpB	1	CTA->TTA	L->L	Silent
68631	G	337	0.991	atpB	2	TCT->TTT	S->F	Conservative
71011	C	6847	0.408	rbcL	3	TCC->TCT	S->S	Silent
71778	C	33	0.485	accD	2	ACG->ATG	T->M	Conservative
72039	C	59	0.780	accD	2	TCA->TTA	S->L	Conservative
72102	C	64	0.437	accD	2	TCC->TTC	S->F	Conservative
72103	C	67	0.373	accD	3	TTC->TTT	F->F	Silent
72603	C	38	0.921	accD	2	TCA->TTA	S->L	Conservative
72773	C	39	0.077	intergenic: 5' of psaI, 3' of accD	–	–	–	–
72774	C	37	0.081	intergenic: 5' of psaI, 3' of accD	–	–	–	–
73077	C	444	0.108	psaI	3	TTC->TTT	F->F	Silent
73121	C	267	0.154	psaI	2	TCC->TTC	S->F	Nonconservative
73124	C	255	0.953	psaI	2	CCA->CTA	P->L	Conservative
73330	C	154	0.227	intergenic: 5' of ycf4, 3' of pasI	–	–	–	–
73334	C	145	0.269	intergenic: 5' of ycf4, 3' of pasI	–	–	–	–
74171	C	50	0.920	ycf4	2	TCA->TTA	S->L	Conservative
74333	C	13	0.231	intergenic: 5' of cemA, 3' of ycf4	–	–	–	–
75258	C	38	0.579	cemA	3	ATC->ATT	I->I	Silent
75533	C	27	1.000	cemA	2	TCC->TTC	S->F	Conservative
75540	C	34	0.118	cemA	3	TTC->TTT	F->F	Silent
75647	C	40	1.000	cemA	2	TCA->TTA	S->L	Conservative
75701	C	21	0.714	cemA	2	CCA->CTA	P->L	Conservative
76326	C	91	1.000	petA	2	TCT->TTT	S->F	Conservative
76678	C	60	0.150	petA	3	CCC->CCT	P->P	Silent
77199	C	37	0.378	intergenic: 3' of petA	–	–	–	–
77427	C	53	0.264	intergenic: 3' of petA	–	–	–	–
77432	C	50	0.320	intergenic: 3' of petA	–	–	–	–
77759	G	462	0.946	psbJ	2	TCA->TTA	S->L	Conservative
77889	G	433	0.085	intergenic: 5' of psbJ	–	–	–	–
78126	G	445	0.967	psbF	2	TCT->TTT	S->F	Conservative
78501	G	351	0.176	intergenic: 5' of psbE	–	–	–	–
78714	G	11	0.364	intergenic: 5' of psbE	–	–	–	–
78781	C	39	0.244	intergenic: 5' of petL	–	–	–	–
78951	C	43	0.756	petL	2	CCA->CTA	P->L	Conservative
78963	C	49	0.804	petL	2	TCA->TTA	S->L	Conservative
78997	C	119	0.529	petL	3	CTC->CTT	L->L	Silent
79231	C	196	0.821	petG	2	TCA->TTA	S->L	Conservative
79431	C	157	0.172	intergenic,3' of petG	–	–	–	–
79551	C	109	0.055	intergenic: 5' of trnW-CCA, 3' of trnP-UGG	–	–	–	–
79553	C	124	0.169	intergenic: 5' of psaJ, 3' of petG	–	–	–	–
79708	C	263	0.076	intergenic: 5' of psaJ	–	–	–	–
80130	C	89	0.584	psaJ	2	TCA->TTA	S->L	Conservative
80378	C	229	0.345	intergenic: 5' of rpl33	–	–	–	–
80383	C	233	0.704	intergenic: 5' of rpl33	–	–	–	–
80855	C	501	0.693	rps18	2	TCG->TTG	S->L	Conservative
80920	C	126	0.651	intergenic: 3' of rps18	–	–	–	–
81225	G	151	0.199	intergenic: 3' of rpl20	–	–	–	–
81283	G	58	0.948	rpl20	2	TCA->TTA	S->L	Conservative
81321	G	54	0.056	rpl20	3	ATC->ATT	I->I	Silent
81359	G	60	0.933	rpl20	1	CAT->TAT	H->Y	Conservative

81413	G	267	0.060	rpl20	1	CGA->TGA	R->*	Nonconservative
81908	G	107	0.121	intergenic: 5' of rpl20	–	–	–	–
81909	G	105	0.638	intergenic: 5' of rpl20	–	–	–	–
82605	G	121	0.116	intergenic: 3' of clpP	–	–	–	–
83134	G	74	0.600	intron: clpPi363	–	–	–	–
83349	G	45	0.143	intron: clpPi363	–	–	–	–
83565	G	134	0.709	clpP	2	TCA->TTA	S->L	Conservative
83691	G	113	0.885	clpP	2	TCA->TTA	S->L	Conservative
83749	G	75	0.067	clpP	1	CAA->TAA	Q->*	Nonconservative
83803	G	72	0.958	clpP	1	CAT->TAT	H->Y	Conservative
83938	G	40	0.085	intron: clpPi71	–	–	–	–
84205	G	56	0.531	intron: clpPi71	–	–	–	–
85179	C	93	0.968	psbB	1	CGG->TGG	R->W	Conservative
85594	C	162	0.981	psbB	2	TCA->TTA	S->L	Conservative
85903	C	154	0.961	psbB	2	TCC->TTC	S->F	Conservative
85904	C	154	0.305	psbB	3	TTC->TTT	F->F	Silent
85906	C	151	0.881	psbB	2	TCC->TTC	S->F	Conservative
85907	C	152	0.743	psbB	3	TTC->TTT	F->F	Silent
85966	C	84	0.988	psbB	2	TCA->TTA	S->L	Conservative
86203	C	424	0.974	psbB	2	TCC->TTC	S->F	Conservative
86204	C	426	0.981	psbB	3	TTC->TTT	F->F	Silent
87105	C	82	0.305	intergenic: 3' of psbT	–	–	–	–
87236	G	351	0.447	psbN	2	TCT->TTT	S->F	Conservative
87264	G	296	0.341	psbN	1	CTA->TTA	L->L	Silent
87268	C	292	0.312	intergenic: 5' of psbH, 3' of psbT	–	–	–	–
87272	G	295	0.444	psbN	2	CCT->CTT	P->L	Conservative
87286	G	289	0.377	psbN	3	TTC->TTT	F->F	Silent
87290	G	276	0.170	psbN	2	ACC->ATC	T->I	Conservative
87571	C	96	0.969	psbH	2	CCG->CTG	P->L	Conservative
87595	C	91	0.956	psbH	2	TCA->TTA	S->L	Conservative
87906	C	343	0.089	intron: petBi6	–	–	–	–
88498	C	174	0.072	intron: petBi6	–	–	–	–
88579	C	745	0.106	petB	3	CCC->CCT	P->P	Silent
88597	C	755	0.824	petB	3	TAC->TAT	Y->Y	Silent
89067	C	357	0.969	petB	2	TCA->TTA	S->L	Conservative
89094	C	267	0.978	petB	2	TCA->TTA	S->L	Conservative
89106	C	231	0.905	petB	2	CCG->CTG	P->L	Conservative
90419	C	272	0.989	petD	2	TCT->TTT	S->F	Conservative
90491	C	123	0.862	petD	2	CCA->CTA	P->L	Conservative
90502	C	49	0.857	petD	1	CAA->TAA	Q->*	Conservative
91457	G	33	0.545	rpoA	2	TCT->TTT	S->F	Conservative
91472	G	40	0.500	rpoA	2	TCA->TTA	S->L	Conservative
91544	G	19	0.842	rpoA	2	TCC->TTC	S->F	Conservative
92638	G	7	0.571	infA	2	GCA->GTA	A->V	Conservative
92850	G	7	0.429	intergenic: 5' of infA, 3' of rps8	–	–	–	–
92965	A	13	0.769	rps8	1	TGT->CGT	C->R	Conservative
93660	G	64	1.000	rpl14	2	CCA->CTA	P->L	Conservative
93806	G	222	0.063	rpl14	3	AAC->AAT	N->N	Silent
94211	G	177	0.051	rpl16	2	TCG->TTG	S->L	Conservative
94311	G	209	0.761	rpl16	1	CAT->TAT	H->Y	Conservative
95606	G	234	0.803	rps3	2	TCA->TTA	S->L	Conservative
96754	G	94	0.052	rps19	2	TCA->TTA	S->L	Conservative
96838	G	99	0.950	rps19	2	TCA->TTA	S->L	Conservative
98443	G	111	0.071	intergenic: 5' of rpl2	–	–	–	–
98643	G	27	0.793	rpl23	2	TCG->TTG	S->L	Conservative
99912	C	22	0.208	tRNA: trnV-GAC	–	–	–	–
109221	G	30	0.433	ndhF	3	CCC->CCT	P->P	Silent
109440	G	48	0.167	ndhF	3	ATC->ATT	I->I	Silent
109908	G	36	0.111	ndhF	3	TTC->TTT	F->F	Silent
109999	G	35	0.943	ndhF	2	CCC->CTC	P->L	Conservative

110336	G	15	0.733	ndhF	1	CGG->TGG	R->W	Conservative
110470	G	17	0.706	ndhF	2	TCA->TTA	S->L	Conservative
110725	G	8	0.875	ndhF	2	ACA->ATA	T->I	Conservative
110747	G	10	0.800	ndhF	1	CGT->TGT	R->C	Conservative
110762	G	11	0.545	ndhF	1	CGG->TGG	R->W	Conservative
110785	G	13	0.308	ndhF	2	ACG->ATG	T->M	Conservative
110801	G	45	0.422	intergenic: 5' of ndhF	–	–	–	–
111299	C	246	0.963	rpl21	2	CCT->CTT	P->L	Conservative
111308	C	236	0.076	rpl21	2	CCC->CTC	P->L	Nonconservative
111682	C	76	0.079	rpl32	2	TCA->TTA	S->L	Nonconservative
112527	C	16	0.312	ccsA	3	GCC->GCT	A->A	Silent
113136	C	49	0.714	ccsA	3	ATC->ATT	I->I	Silent
113174	C	75	0.627	ccsA	2	TCA->TTA	S->L	Conservative
113333	C	46	0.957	ccsA	2	TCA->TTA	S->L	Conservative
113773	G	13	0.923	ndhD	2	ACA->ATA	T->I	Conservative
113814	G	39	0.103	ndhD	3	TCC->TCT	S->S	Silent
113857	G	43	0.953	ndhD	2	TCA->TTA	S->L	Conservative
114700	G	19	0.947	ndhD	2	TCG->TTG	S->L	Conservative
114707	G	19	0.947	ndhD	1	CAT->TAT	H->Y	Conservative
114754	G	33	0.970	ndhD	2	CCA->CTA	P->L	Conservative
114991	G	18	0.778	ndhD	2	TCA->TTA	S->L	Conservative
114994	G	17	0.882	ndhD	2	TCA->TTA	S->L	Conservative
115003	G	24	0.833	ndhD	2	CCT->CTT	P->L	Conservative
115048	G	13	0.231	ndhD	2	ACG->ATG	T->M	Conservative
115157	G	69	0.130	intergenic: 5' of ndhD, 3' of psaC	–	–	–	–
115530	G	57	0.193	ndhE	2	ACA->ATA	T->I	Conservative
115646	G	17	0.235	ndhE	3	CTC->CTT	L->L	Silent
116216	G	41	0.927	ndhG	2	TCG->TTG	S->L	Conservative
116243	G	33	0.848	ndhG	2	TCA->TTA	S->L	Conservative
116252	G	28	0.107	ndhG	2	GCA->GTA	A->V	Nonconservative
116288	G	45	0.867	ndhG	2	CCG->CTG	P->L	Conservative
116384	G	108	0.843	ndhG	2	TCG->TTG	S->L	Conservative
116953	G	57	0.053	ndhI	3	CAC->CAT	H->H	Silent
117333	G	52	0.962	ndhA	2	CCA->CTA	P->L	Conservative
117354	G	39	0.872	ndhA	2	TCA->TTA	S->L	Conservative
117627	G	184	0.768	ndhA	2	CCA->CTA	P->L	Conservative
117628	G	196	0.102	ndhA	1	CCA->TTA	L->L	Silent
117796	G	38	0.947	intron: ndhAi556	–	–	–	–
118570	G	22	0.909	ndhA	2	ACT->ATT	T->I	Conservative
118771	G	28	0.786	ndhA	2	GCT->GTT	A->V	Conservative
119092	G	26	0.346	ndhA	2	ACG->ATG	T->M	Conservative
119299	G	56	0.161	ndhH	3	TTC->TTT	F->F	Silent
119321	G	76	0.842	ndhH	2	TCT->TTT	S->F	Conservative
119393	G	58	0.914	ndhH	2	CCT->CTT	P->L	Conservative
119408	G	112	0.964	ndhH	2	TCA->TTA	S->L	Conservative
119606	G	79	0.785	ndhH	2	TCA->TTA	S->L	Conservative
119777	G	100	0.900	ndhH	2	TCA->TTA	S->L	Conservative
119846	G	34	0.824	ndhH	2	CCA->CTA	P->L	Conservative
119880	G	19	0.947	ndhH	1	CAC->TAC	H->Y	Conservative
121173	G	99	0.980	ycf1	2	TCC->TTC	S->F	Nonconservative
121344	G	78	1.000	ycf1	2	TCA->TTA	S->L	Conservative
121626	G	85	0.082	ycf1	2	TCT->TTT	S->F	Conservative
121785	G	22	0.182	ycf1	2	ACC->ATC	T->I	Conservative
122092	G	48	0.771	ycf1	1	CAT->TAT	H->Y	Conservative
123849	G	10	1.000	ycf1	2	ACT->ATT	T->I	Conservative
125017	A	3	1.000	ycf1	1	TGA->CGA	*->R	Conservative
125361	G	5	0.800	ycf1	2	ACA->ATA	T->I	Nonconservative
125852	G	22	0.182	intergenic: 5' of ycf1, 3' of chlN	–	–	–	–
126000	G	81	0.988	chlN	2	TCT->TTT	S->F	Conservative
126039	G	107	1.000	chlN	2	TCA->TTA	S->L	Conservative

126734	G	134	0.052	chlN		3	TTC->TTT	F->F	Silent
126846	G	130	0.985	chlN		2	TCA->TTA	S->L	Conservative
127184	G	69	0.058	chlN		3	TGC->TGT	C->C	Silent
127337	G	61	1.000	chlL		1	CGA->TGA	R->*	Conservative

Table S3B. Plastid editing locations for *Psilotum nudum*

Genome Position	Ref Base	RNA Coverage	Editing Efficiency	Location	Codon Position	Codon Change	AA Change	Edit Site Class
9908	G	74	0.243	matK	1	CAT->TAT	H->Y	Conservative
13920	G	1829	0.324	psaM	2	TCG->TTG	S->L	Conservative
14292	G	1229	0.346	intergenic: 5' of psaM	–	–	–	–
24735	G	4	1.000	rpoC2	1	CGC->TGC	R->C	Conservative
29746	G	12	0.583	rpoB	2	CCG->CTG	P->L	Conservative
30898	G	13	0.615	rpoB	2	CCA->CTA	P->L	Conservative
32919	G	1135	0.206	psbM	2	CCT->CTT	P->L	Conservative
32946	G	1283	0.326	psbM	2	TCA->TTA	S->L	Conservative
36028	G	1643	0.256	psbC	3	ACC->ACT	T->T	Silent
40112	G	282	0.834	rps14	2	TCA->TTA	S->L	Conservative
41452	G	58	0.051	psaB	3	TTC->TTT	F->F	Silent
50674	G	81	0.573	ndhJ	2	TCG->TTG	S->L	Conservative
58102	C	31	0.839	accD	2	TCA->TTA	S->L	Conservative
63105	C	258	0.213	petA	1	CTT->TTT	L->F	Conservative
66150	C	181	0.635	petG	2	TCA->TTA	S->L	Conservative
70172	G	290	0.749	clpP	2	TCA->TTA	S->L	Conservative
70657	G	238	0.529	intron: clpPi71	–	–	–	–
76147	C	401	0.930	petD	2	TCA->TTA	S->L	Conservative
82168	G	107	0.682	rpl22	2	TCA->TTA	S->L	Conservative
86316	G	496	0.663	ndhB	2	CCA->CTA	P->L	Conservative
87919	G	76	0.767	ndhB	2	CCA->CTA	P->L	Conservative
88942	G	514	0.844	rps7	2	TCG->TTG	S->L	Conservative
101092	G	57	0.053	ndhF	3	CCC->CCT	P->P	Silent
101148	G	10	0.400	ndhF	1	CGG->TGG	R->W	Conservative
101432	G	60	0.367	ndhF	2	CCA->CTA	P->L	Conservative
102412	C	64	0.092	intergenic: 5' of rpl32, 3' of rpl21	–	–	–	–
104679	C	72	0.917	ccsA	2	TCT->TTT	S->F	Conservative
